# Effect on intensity of treadmill running on learning, memory and expressions of cell cycle-related proteins in rats with cerebral ischemia

**DOI:** 10.18632/oncotarget.16537

**Published:** 2017-03-24

**Authors:** Ya-Ning Zhao, Jian-Min Li, Chang-Xiang Chen, Shu-Xing Li, Cheng-Jing Xue

**Affiliations:** ^1^ Nursing and Rehabilitation College, North China University of Science and Technology, 063000, China; ^2^ The Neurosurgery of Affiliated Hospital, North China University of Science and Technology, 063000, China

**Keywords:** cerebral ischemia, treadmill running, learning and memory, cyclin A, cyclin E

## Abstract

**Objective:**

We discussed the intensity of treadmill running on learning, memory and expression of cell cycle-related proteins in rats with cerebral ischemia.

**Method:**

Eighty healthy male SD rats were randomly divided into normal group, model group, intensity I group and intensity II group, with 20 rats in each group. The four-vessel occlusion method of Pulsinelli (4-VO) was used to induce global cerebral ischemia. Brain neuronal morphology was observed by hematoxylin-eosin (HE) staining at 3h, 6h, 24h and 48h after modeling, respectively. Hippocampal expressions of cyclin A and cyclin E were detected by immunohistochemistry. At 48h after modeling, the learning and memory performance of rats was tested by water maze experiment.

**Result:**

Compared with the normal group, the other three groups had a significant reduction in surviving neurons, prolonging of escape latency and decreased number of passes over the former position of the platform (P<0.05). The number of surviving neurons and the number of passes over the former position of the platform were obviously lower in the model group than in intensity I group (P<0.05), but significantly higher compared with intensity II group (P<0.05). Escape latency of the model group was obviously prolonged as compared with intensity I group (P<0.05), but much shorter than that of intensity II group (P<0.05). Compared with the normal group, the expressions of cyclin A and cyclin E were significantly upregulated at different time points after modeling (P<0.05). The expression of the model group was higher than that of intensity I group, but lower than that of intensity II group (P<0.05).

**Conclusion:**

Moderate intensity of treadmill running can help protect brain neurons and improve learning and memory performance of rats with global cerebral ischemia. But high intensity of treadmill running has a negative impact, possibly through the regulation of cell cycle-related proteins in ischemia/reperfusion injury.

## INTRODUCTION

Ischemic cerebrovascular disease has a high incidence, high mortality and high disability rate, posing great threat to human health [1] and leading to movement disorder and decline in learning performance [2]. Hippocampus is an important part of learning and memory of high-level nervous activity. Recent studies have shown that hippocampal neuron loss has a close relationship with cognitive dysfunction. Physical exercise is considered to be conducive to the recovery of brain functions after ischemia. Study [3–4] has shown that physical exercise can promote vessel and nerve regeneration, inhibit cell apoptosis and neurogenic inflammation and improve learning capacity. Aerobic exercise offers major rehabilitation benefits for cerebral ischemia [5–6], improving functional disorders and patient's life quality. But persistent, high-intensity exercise will aggravate lipid peroxidation, protein oxidation and cell apoptosis [7]. Treadmill running is a form of aerobic exercise. Liu et al [8] performed a study on the treadmill exercise on young rats’ nerve regeneration and found that the amount of treadmill exercise can significantly promote hippocampal nerve regeneration in young and different life-stage rats.

Cell cycle is defined as the period between successive divisions of a cell and consists of G1, S, G2 and M phase [9]. Cyclin E is mainly expressed in G1/S phase, and cyclin A is crucial for DNA replication in S phase. Under ischemic and hypoxic conditions, neurons will re-enter a cell cycle for DNA repair [10]. It is still uncertain which intensity of treadmill running is most appropriate for neuronal regeneration after ischemia and whether this effect is associated with cell cycle regulation. We built the ischemia/reperfusion model in rats to study the effect of treadmill running on spatial learning, memory and expression of cell cycle-related proteins. Finally, we tentatively propose a molecular mechanism to explain the result.

## RESULTS

### The effect of treadmill running on brain neurons of rats between the groups

**Figure 1:**
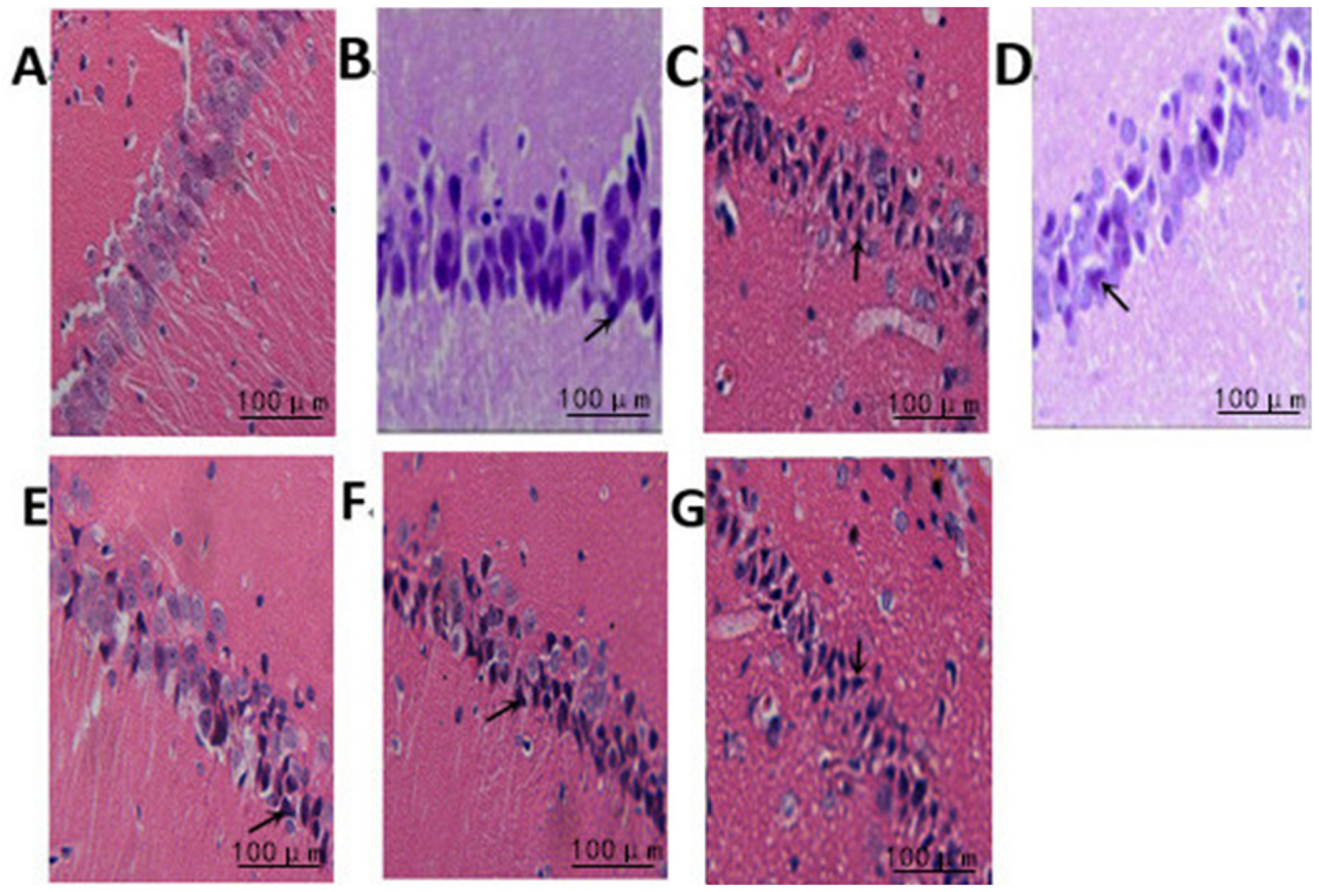
The effect of treadmill running on brain neurons of rats between the groups (400×) Note: **(A)** Normal group; **(B)** Model group at 6h; **(C)** Model group at 48h; **(D)** IntensityIgroup at 6h; **(E)** IntensityIgroup at 48h; **(F)** IntensityII group at 6h; **(G)** IntensityII group at 48h. The arrow shows the dead nerve cells.

Hippocampal neurons in the normal group showed regular arrangement with intact nuclei and distinct nucleoli. The nuclei were deeply stained and showed pyknosis in the model group, and the nuclei were lost in some neurons with irregular arrangement. In intensity I group, some neurons were intact with less serious pyknosis. In intensity II group, the neurons were seriously damaged and necrotic, with pyknosis and obscure boundary between nuclei and cytoplasm. Compared with the normal group, the other three groups displayed a significant reduction in surviving neurons in the hippocampus at all time points (3h, 6h, 24h, 48h) (P<0.05). The survival rate of the model group was significantly lower than that of intensity I group (P<0.05) and higher than that of intensity II group (P<0.05) (Figure 1, Table 1).

**Table 1:** Comparison of percentage of surviving hippocampal neurons in each group (x¯±s, %)

Group	*n*	3h	6h	24h	48h
Normal group	5	97.67 ± 1.53	98.33 ± 1.53	97.67 ± 1.52	97.67 ± 1.52
Model group	5	83.67 ± 1.52^①^	76.33 ± 1.50^①^	69.00 ± 1.00^①^	60.22 ± 2.13^①^
Intensity I group	5	90.33 ± 1.54^①②^	84.67 ± 1.53^①②^	77.65 ± 1.32^①②^	70.23 ± 2.02^①②^
Intensity II group	5	75.53±1.53^①②③^	69.86±1.52^①②③^	61.47±1.22^①②③^	52.36±2.08^①②③^
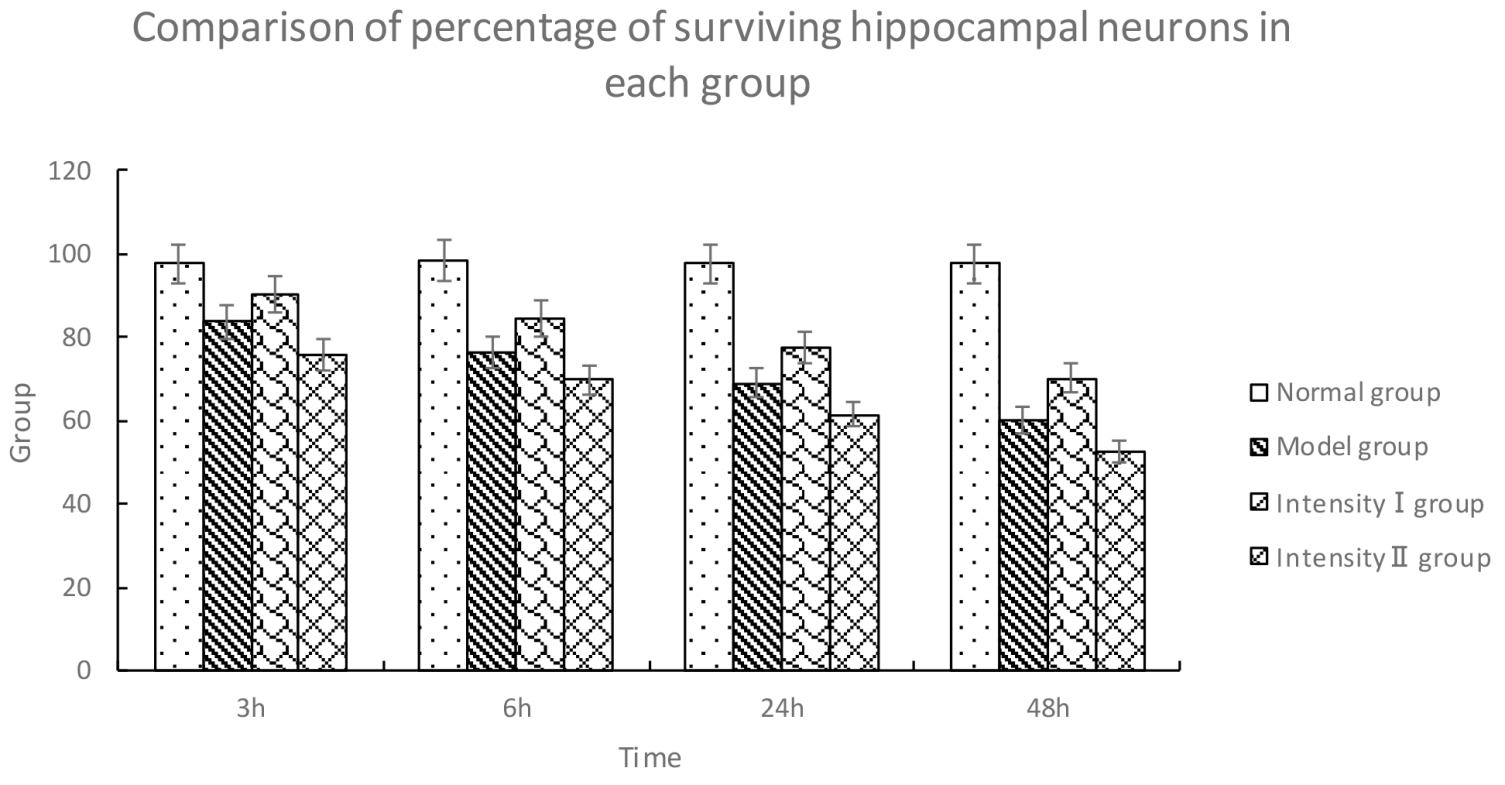					

### The effect of treadmill running on spatial learning performance of rats between the groups

**Figure 2:**
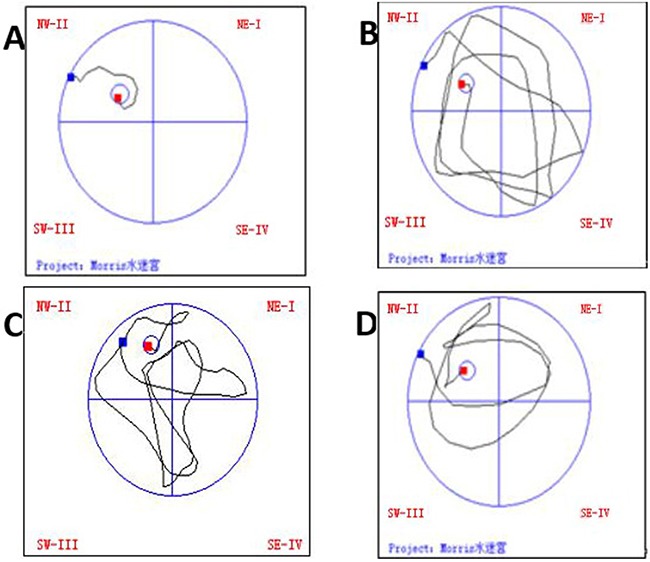
The effect of treadmill running on trajectories in the water maze of rats in each group Note: **(A)** Normal group; **(B)** Model group at 48h; **(C)** IntensityIgroup at 48h; **(D)** Intensity II group at 48h.

Compared with normal group, the other three groups had obviously prolonged latency and much less passes over the former position of platform (P<0.05). Model group had significantly prolonged latency and much less passes over the former position of platform as compared with intensity I group (P<0.05); however, the latency was obviously shortened as compared with intensity II group, with less passes over the former position of platform (P<0.05) (Figure 2, Table 2).

**Table 2:** Results of water maze test in each group (x¯±s)

Group	n	Latency	Number of passes
Normal group	5	9.08 ± 2.64	11.90 ± 1.45
Model group	5	33.08 ± 5.85^①^	4.00 ± 0.67^①^
Intensity I group	5	24.20 ± 4.66^①②^	6.30 ± 1.16^①②^
Intensity II group	5	44.12±4.98^①②③^	2.01±1.06^①②③^
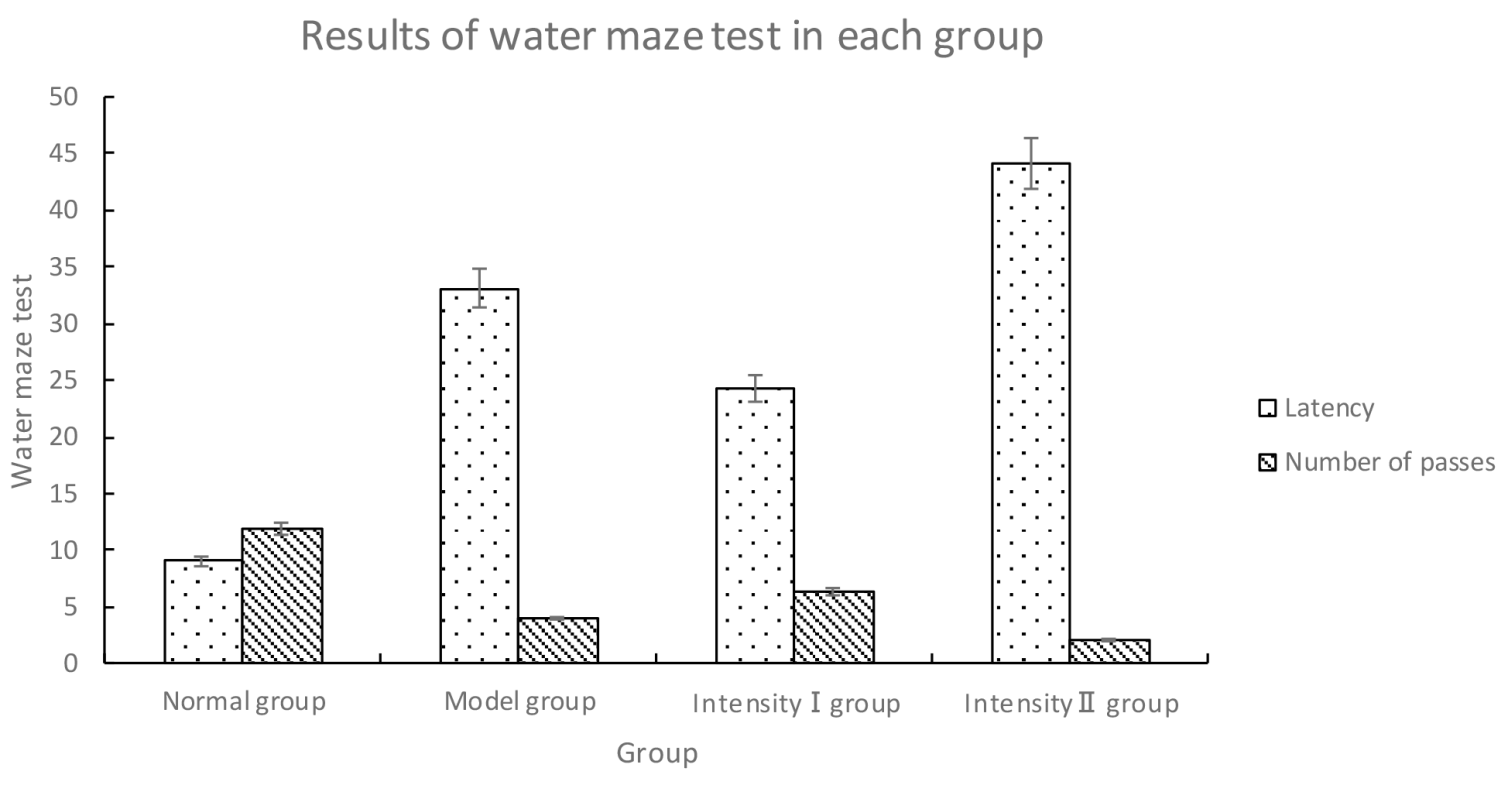				

### The effect of treadmill running on hippocampal expressions of cyclin A and cyclin E in each group

#### Immunohistochemical analysis of cyclin A

**Figure 3:**
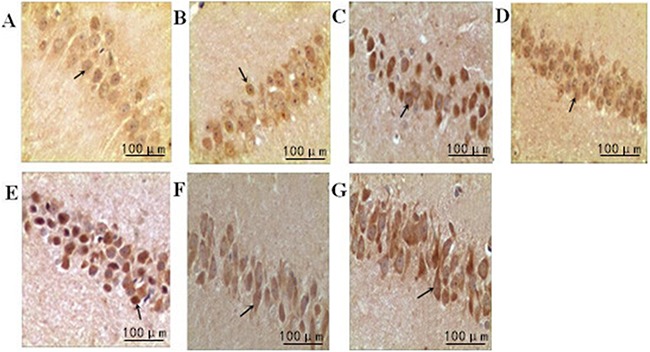
The effect of treadmill running on hippocampal expressions of cyclin A in each group (400×) Note: **(A)** Normal group; **(B)** Model group at 6h; **(C)** Model group at 48h; **(D)** Intensity I group at 6h; **(E)** Intensity I group at 48h; **(F)** Intensity II group at 6h; **(G)** IntensityII group at 48h. The arrow shows immune positive nerve cell.

The cells positive for cyclin A were stained brown in the nuclei. Compared with the normal group, the expression of cyclin A was gradually increased over time in the model group (P<0.05). Compared with the model group, the expression of cyclin A in intensity I group decreased over time (P<0.05); in contrast, the expression of cyclin A in intensity II group increased significantly (P<0.05) (Figure 3, Table 3).

**Table 3:** Comparison of number of cyclin A-positive cells in each group (x¯±s, cells/high-power field of vision)

Group	*n*	3h	6h	24h	48h
Normal group	5	6.20 ± 0.42	6.20 ± 0.42	6.40 ± 0.43	6.40 ± 0.43
Model group	5	8.40 ± 0.52^①^	11.70 ± 1.06^①^	15.50 ± 0.53^①^*	22.40 ± 0.52^①^
Intensity I group	5	7.89 ±0.43^①②^	10.67 ±1.01^①②^	13.42 ±0.48^①②^	20.65 ±0.41^①②^
Intensity II group	5	10.60 ± 0.84^①②③^	16.70 ± 0.68^①②③^	24.50 ± 0.53^①②③^	36.20 ± 1.40^①②③^
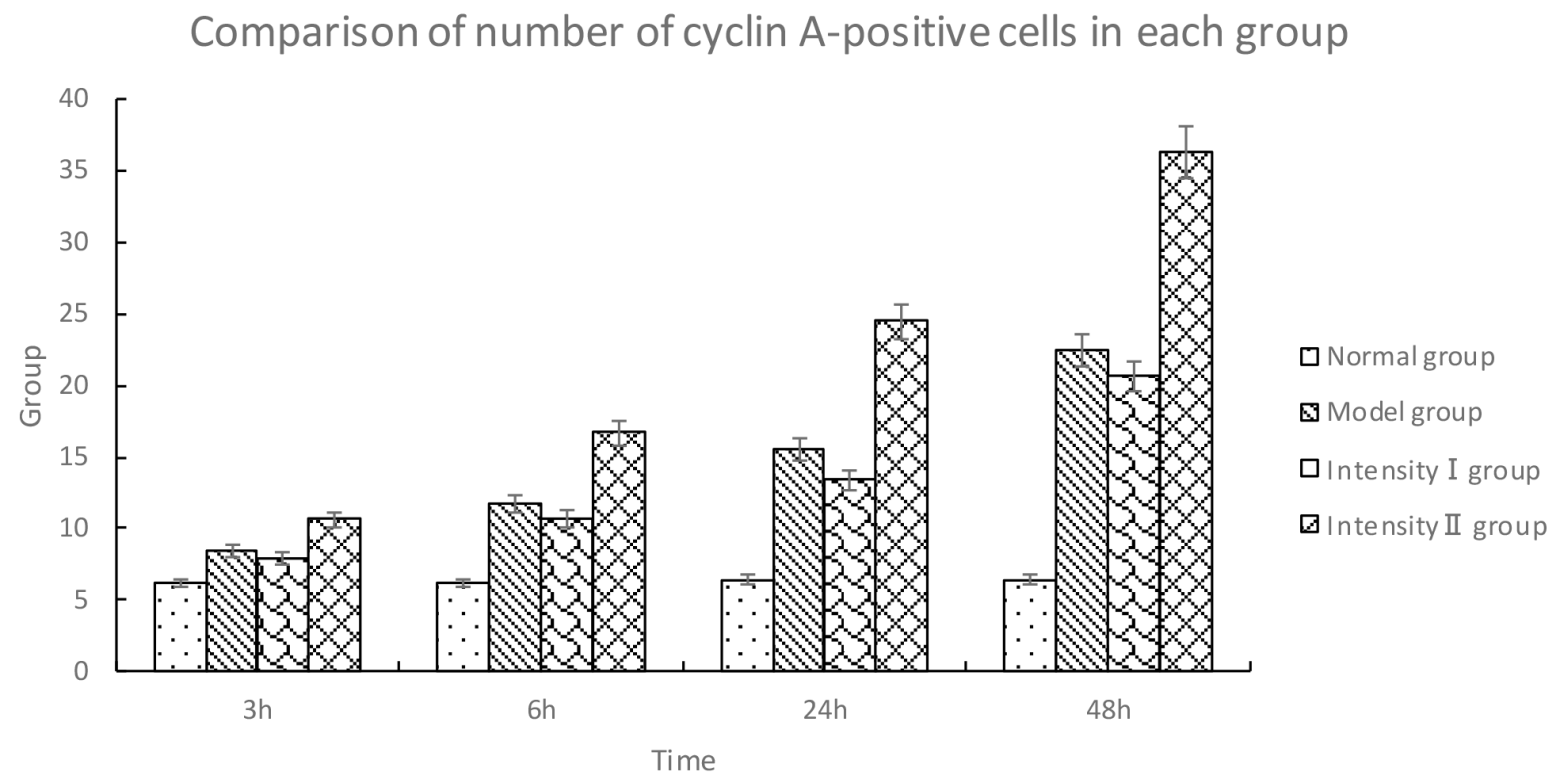					

#### Immunohistochemical analysis of cyclin E

**Figure 4:**
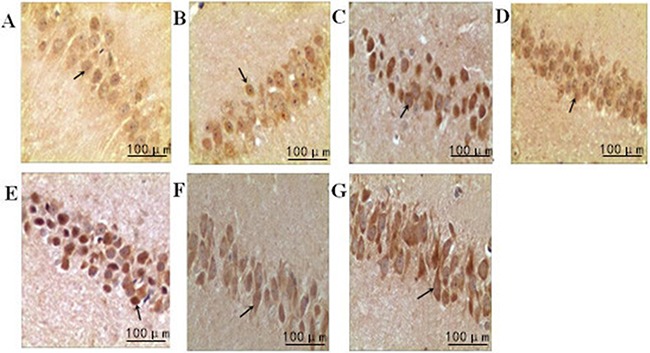
The effect of treadmill running on hippocampal expressions of cyclin E in each group (400×) Note: **(A)** Normal group; **(B)** Model group at 6h; **(C)** Model group at 48h; **(D)** Intensity I group at 6h; **(E)** IntensityI group at 48h; **(F)** Intensity II group at 6h; **(G)** IntensityII group at 48h. The arrow shows immune positive nerve cell.

The cyclin E-positive cells were stained brown in the nuclei. Compared with the normal group, the model group showed a significant upregulation of cyclin E over time (P<0.05). Compared with the model group, intensity I group had a downregulation of cyclin E at each time point (P<0.05); however, the expression of cyclin E in intensity II group was significantly upregulated over time (P<0.05) (Figure 4, Table 4).

**Table 4:** Comparison of number of cyclin E-positive cells in each group (x¯±s, cells/high-power field of vision)

Group	*n*	3h	6h	24h	48h
Normal group	5	7.90 ± 0.74	7.80 ± 1.23	7.50 ± 1.27	7.50 ± 1.27
Model group	5	20.30 ± 0.48^①^	15.20 ± 0.63^①^	10.00 ± 0.82^①^	7.70 ± 0.68^①^
Intensity I group	5	16.42±0.53^①②^	11.36±0.58^①②^	8.65±0.72^①②^	6.90±0.45^①②^
Intensity II group	5	31.60 ± 0.70^①②③^	24.50 ± 0.70^①②③^	16.80 ± 0.63^①②③^	9.10 ± 0.74^①②③^
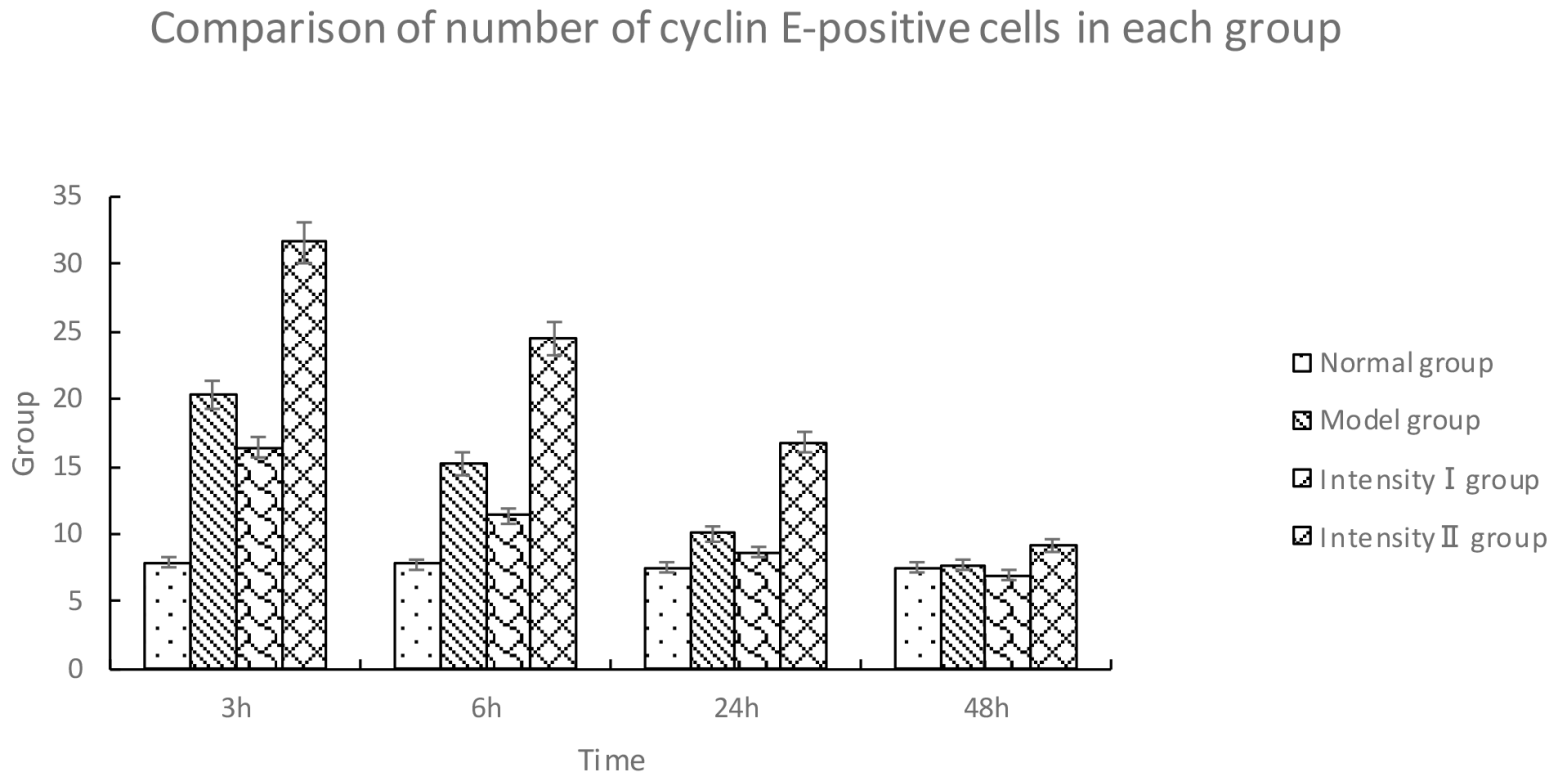					

## DISCUSSION

After induction of global cerebral ischemia, hippocampal neurons of the model group showed degenerative necrosis and irregular arrangement by HE staining, prolonged latency and lower number of passes over the former position of the platform in water maze test. This indicated neuronal damage and spatial memory disorder after cerebral ischemia. Compared with the model group, intensity I group showed less severe interstitial edema, pyknosis and vacuole formation, indicating improvement of spatial learning and memory performance after treadmill running at intensity I. But at intensity II, the hippocampal neurons showed irregular morphology with a marked decline in spatial learning and memory. According to the result, treadmill running at intensity II aggravated cerebral ischemia and hypoxia, inducing neurological deficit, irreversible damage and further decline of spatial learning and memory performance. Thus treadmill running at moderate intensity can improve neuronal damage caused by ischemia, while high intensity had a negative impact. This is consistent with the findings by Liu, Sun and Shen et al. [15–17]. Physical exercise of varying intensity tends to have different impact on the nerve system. For example, K. Kamijo [18] found that high-intensity exercise led to a decline of P300 amplitude, while moderate-intensity exercise caused it to increase and low-intensity exercise did not bring about obvious changes. Wang et al. [19] administered aerobic exercise of moderate to high intensity to patients with Alzheimer's disease and found that both intensity levels improved the cognitive functions and mental symptoms of patients. We also found that moderate intensity was superior to high intensity in improving spatial learning and memory of rats with cerebral ischemia. Instead of promoting neuroprotective, high-intensity treadmill running further impaired the cognitive functions. An important inspiration drawn from the study is that an appropriate intensity should be chosen for rehabilitation exercise.

According to our results, hippocampal neurons in the model group showed degenerative necrosis and irregular arrangement, and cyclin E expression first increased and then decreased; cyclin A expression gradually increased over time. Brain neuronal damage caused an upregulation of cyclin E, and as the damaged neurons entered S phase from G_0_/G_1_ phase, cyclin E expression decreased, while cyclin A expression increased. In spite of the stress-induced defense mechanism of the brain, neuronal damage occurred, causing a decline in spatial learning and memory capacity in water maze test. In intensity I group, the variation of cyclin E and cyclin A over time was consistent with that of the model group, and a downregulation of the two proteins in intensity I group indicated an improvement of brain functions. But for intensity II group, the expressions of cyclin E and cyclin A were significantly upregulated as compared with the model group, indicating deterioration of brain functions. The differences in protein expressions reached the significant level between the two intensities. It can be inferred that moderate intensity of treadmill running promotes neural repair by regulating cell cycle-related proteins, while high intensity causes further damage. Mature neurons do not differentiate or proliferate, and are arrested in G_0_ phase. Normal neurons contain very small amount of cyclin A and cyclin E. Recent study shows that the neurons will re-enter the cell cycle in response to ischemic and hypoxic conditions, which offers self-defense for the damaged cells. For example, according to some studies [20–21], cell cycle-related proteins are upregulated in case of cerebral ischemia or neurodegenerative disorders, which is accompanied by a decline in learning, memory and sensorimotor capacity. Yao et al. [22] performed injection of Aβ_1-40_ into the hippocampus of rats to induce dementia. It was found that cyclin A was upregulated as compared with the normal group, indicating impaired cognition. After injection of *Gynostemma pentaphyllum*, the demented rats showed an obvious cognitive improvement with a change in cyclin A expression. Our preliminary experiment also indicated the favorable effect of treadmill running on ischemia-induced cognitive deficit by regulating cell cycle-related proteins. But moderate and high intensity of treadmill running had opposite effect in the present study, which may be due to different effect on neurotrophic factors acting on the cell cycle-related proteins under two intensities. In other researches [23–24], the serum level of brain-derived neurotrophic factors increased at 60% of maximum oxygen uptake, but it rapidly declined at high intensity exercise. Gomez [25] showed that wheel running upregulated the expressions of neurotrophic factors in rats with stroke and promoted the recovery of motor functions. Soya H [26] administered different intensity of treadmill running to rats and only low intensity promoted the expressions of neurotrophic factors. Therefore, we speculated that the neurotrophic factors were downregulated under high intensity, which affected the expressions of cell cycle-related proteins and hence the learning and memory capacity and neuronal survival of rats.

To conclude, moderate intensity of physical exercise has a neuroprotective effect in rats with cerebral ischemia.

Exercise training, as a non-drug therapy, has attracted considerable attention in the field of neural rehabilitation, and is very important in clinical rehabilitation exercise on patients with stroke. If the exercise with appropriate intensity was under the supervision and guidance in brain injury patient, the incidence of the cognitive disorder after stroke will be significantly reduced and the life quality of stroke patients will be improved significantly. While this study found high intensity aggravates neuronal damage. Thus for patients with cerebral ischemia, high-intensity rehabilitation exercise should be avoided.

## MATERIALS AND METHODS

### Animals and reagents

**Figure 5:**
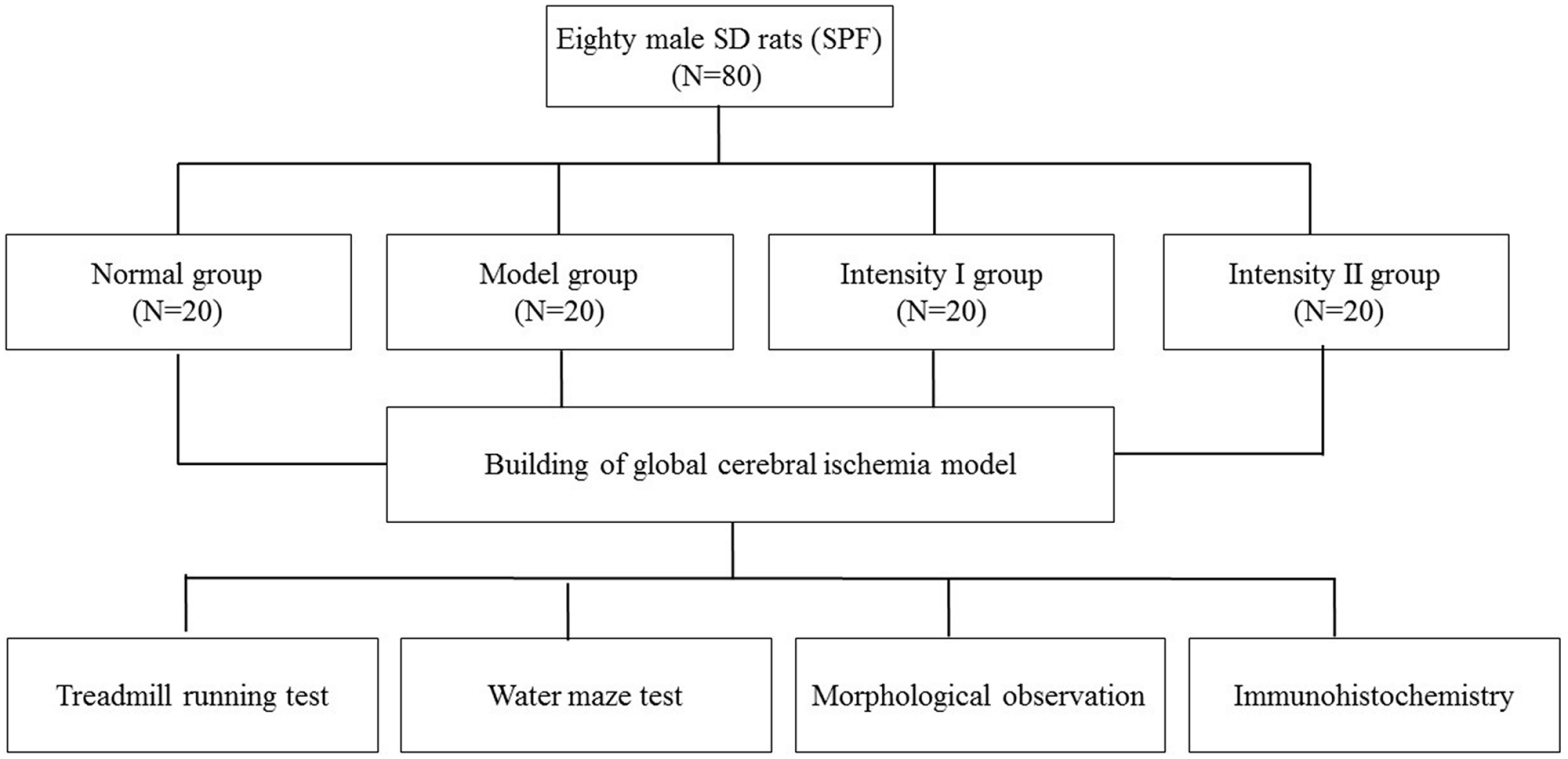
Experimental design and grouping

Eighty male SD rats (SPF) weighing 250-300g were purchased from Beijing Huafukang Biotechnology Co., Ltd. (license No.: SCXK (Beijing) 2009-0004). The rats were randomly divided into normal group, model group, intensity I group and intensity II group, with 20 rats in each group. Four time points were set for each group (3h, 6h, 24h and 48h), with 5 rats tested in each group at each time point (As shown in Figure 5). The room temperature was controlled at 23±2°C, with natural illumination. Rabbit anti-rat antibodies against cyclin A and cyclin E were purchased from Beijing Biosynthesis Biotechnology Co., Ltd. DAB substrate kit, PBS buffer and citrate buffer were purchased from Beijing Zhongshan Golden Bridge Biotechnology Co., Ltd. Equipments used were as follows: electrocoagulator (Department of Neurosurgery, Affiliated Hospital of North China University of Science and Technology), ZH-PT treadmill running deck (Huaibei Zhenghua Biological Apparatus Co., Ltd.), optical microscope (Olympus, Japan), microtome (Leica, Germany), and constant-temperature incubator (Hirasawa).

### Materials and Methods

#### Building of global cerebral ischemia model

The four-vessel occlusion method of Pulsinelli (4-VO) was used to produce global cerebral ischemia/reperfusion model in rats [11]. The rats were fasted from food for 8h before surgery but allowed access to water. After anesthesia by injection of 10% chloral hydrate, bilateral vertebral arteries deep in the bilateral smaller pterygoid foramen were electrocoagulated to induce complete occlusion. The rats were fixed in a supine position on the surgery table. Common carotid artery was dissociated and the thread was inserted on both sides. When the rats awoke from aesthesia 24h later, bilateral common carotid arties were clipped for 15min. For the normal group, vertebral arteries and common carotid arteries were fully exposed but not clipped.

#### Treadmill running test [12]

The rats were adapted to the treadmill by running on it at the incline of 0°at 9:00 for 7 days. The speed was set as 10m/min and 20m/min for 10min, respectively, and the exercise stopped after the rats ran on the treadmill at 20m/min for 30min (equivalent to 30% of maximum oxygen uptake VO2max). This speed and duration were used for rats in intensity I group. For intensity II group, the speed was increased to 19.5m/min within 3min starting from 10m/min (equivalent to 70% of maximum oxygen uptake VO2max). The test did not stop until exhaustion, which was defined as the conditions [13] of strenuity and semireclining position in running, no recovery of physical strength after reduction of speed, shortness of breath, tiredness, and slow reaction to external stimuli.

#### Water maze test

Morris water maze test was performed [14] at 48h after modeling. Five rats were selected from each group and trained to find a hidden platform twice daily for 3 days. The platform was placed in fixed position in the four quadrants, respectively. The longest length of swimming was 90s and the latency to reach the hidden platform was recorded. The platform was removed on the fourth day, and the number of passes over the position of former platform was measured.

#### Morphological observation

Five rats were selected from each group at each time point. Brain tissues were harvested after perfusion using 4% paraformaldehyde. HE staining was performed from optic chiasma to transverse cerebral fissure, followed by conventional paraffin embedding. Leica microtome was used to make coronal sections (thickness 4μm), which were toasted at 60°C in the oven for 24h. After dewaxing in xylene, the sections were dehydrated with an ethanol gradient (100%, 95%, 80%). The sections were counterstained with hematoxylin, differentiated in 1% hydrochloric acid in ethanol, and washed with running water to return the nuclei to blue. Next the procedures of counterstaining with 0.5% eosin, ethanol gradient dehydration (80%, 95% and 100%), transparentization in dimethylbenzene and sealing with neutral balsam were performed routinely. The sections were observed under the optical microscope. The number of survival neurons in each field of vision was counted (400×), and the images were collected and analyzed by Motic-6.0 software. The average percentage of surviving neurons in each field of vision was calculated (%).

#### Immunohistochemistry

The sections were dewaxed and hydrated, and citrate buffer antigen retrieval was performed. The sections were incubated with primary antibodies at 4°C overnight. After washing with PBS, secondary antibodies were added and the cells were incubated at 37°C in an incubator for 40min. DAB substrate was added for color development. When brown particles appeared under the microscope, color development was terminated by washing with running water. As with HE staining, counterstaining with hematoxylin, differentiation in hydrochloric acid, transparentization in dimethylbenzene and sealing with neutral balsam were performed routinely. For each rat 4 slides were observed under the optical microscope.

#### Statistical analysis

The data were input into Excel 2003 and reported as x¯±s. Repeated measures one-way ANOVA was carried out using SPSS17.0 software, and t-test was used for pairwise comparisons. P<0.05 indicated significant difference.
